# Robust singlet fission in pentacene thin films with tuned charge transfer interactions

**DOI:** 10.1038/s41467-018-03300-1

**Published:** 2018-03-05

**Authors:** K. Broch, J. Dieterle, F. Branchi, N. J. Hestand, Y. Olivier, H. Tamura, C. Cruz, V. M. Nichols, A. Hinderhofer, D. Beljonne, F. C. Spano, G. Cerullo, C. J. Bardeen, F. Schreiber

**Affiliations:** 1 Institute of Applied Physics and Center for Light Matter Interactions, Sensors and Analytics, LISA+, University of Tübingen, Auf der Morgenstelle 10, 72076 Tübingen, Germany; 2 Fritz-Haber Institute of the Max-Planck Society, Faradayweg 4-6, 14195 Berlin, Germany; 3 IFN-CNR, Dipartimento di Fisica, Politecnico di Milano, Milano, 20133 Italy; 4 Department of Chemistry, Temple University, Philadelphia, PA 19122 USA; 5 Laboratory for Chemistry of Novel Materials, University of Mons, Place du Parc 20, 7000 Mons, Belgium; 6 Department of Chemical System Engineering, The University of Tokyo, 7-3-1 Hongo Bunkyo-ku, Tokyo, 113-8656 Japan; 7 Department of Chemistry, University of California at Riverside, 501 Big Springs Rd, Riverside, CA 92521 USA; 8 School of Chemistry and Biochemistry and Center for Organic Photonics and Electronics, Georgia Institute of Technology, Atlanta, GA 30332-0400 USA

## Abstract

Singlet fission, the spin-allowed photophysical process converting an excited singlet state into two triplet states, has attracted significant attention for device applications. Research so far has focused mainly on the understanding of singlet fission in pure materials, yet blends offer the promise of a controlled tuning of intermolecular interactions, impacting singlet fission efficiencies. Here we report a study of singlet fission in mixtures of pentacene with weakly interacting spacer molecules. Comparison of experimentally determined stationary optical properties and theoretical calculations indicates a reduction of charge-transfer interactions between pentacene molecules with increasing spacer molecule fraction. Theory predicts that the reduced interactions slow down singlet fission in these blends, but surprisingly we find that singlet fission occurs on a timescale comparable to that in pure crystalline pentacene. We explain the observed robustness of singlet fission in such mixed films by a mechanism of exciton diffusion to hot spots with closer intermolecular spacings.

## Abstract (web summary)

Singlet fission is desirable for highly efficient solar cells, yet the details of the process remain elusive. Here, Broch et al. show that the changes in bulk intermolecular interactions can have little effect on the singlet fission rate in a mixture of pentacene and weakly interacting spacer molecules.

## Introduction

Intermolecular interactions, including the full or partial transfer of charge, are not only relevant for the performance of organic semiconductors (OSCs)^1,2^ and their blends in devices, but are also of fundamental importance to obtain a better understanding of OSC photophysics, ranging from steady state absorption spectra^3–6^ to complex dynamical processes such as singlet fission (SF)^7^. The latter is a spin-conserving way to convert one high-energy singlet exciton into a pair of lower energy triplet excitons and is receiving strong attention as a way to increase solar cell efficiency^7,8^. The design of new materials that can undergo SF with high efficiency is a challenge because a predictive understanding of how intermolecular interactions affect this nonradiative relaxation pathway is still lacking^3,4,6^. One way to obtain such an understanding is to continuously tune the aggregation properties between the limits of isolated molecule and close-packed crystal. There are appealing approaches to modify the strength of intermolecular interactions using chemical modifications of molecules^9–13^ or nanoparticles^14^, but these methods are faced with several difficulties including limited flexibility in material choice or additional unpredictable effects of molecular arrangement and orientation. The impact of molecular packing on SF has been demonstrated for example by Bradforth and co-workers for 5,12-diphenyl tetracene (DPT), which does not exhibit SF in its single crystalline form, but, nevertheless displays SF in amorphous films^15^. There, the increased disorder leads to various packing geometries and intermolecular distances, some of which are favorable for SF. Furthermore, for derivatives of the prototypical OSC pentacene (PEN, C_22_H_14_), which is a highly efficient SF material^9,16,17^, the SF rate is measured to change by a factor of 100 depending on the specific molecular structures and packing^18–20^.

Here we report a highly controllable way to tune intermolecular interactions in thin films namely by blending the OSC with weakly interacting spacer molecules. The molecular ratio of the OSC of interest and the spacer molecule can be conveniently chosen and precisely controlled during sample preparation. This method does not require any chemical modification and, importantly, does not strongly affect the molecular arrangement of the OSC in the thin films. By systematically reducing intermolecular interactions without a concomitant change in the average molecular packing motif, we can determine how much a neat crystal can be modified before its electronic dynamics, e.g. the SF rate, change significantly. As proof of principle, we chose PEN, which allows us to follow an approach opposite to Bradforth et al.^15^, by starting with a material which exhibits SF in single crystalline form (i.e., with well-defined intermolecular distances) and  attempting to reduce the SF efficiency by decreasing the strength of intermolecular interactions.

We mix PEN with two different spacer molecules, diindenoperylene (DIP, C_32_H_16_) and picene (PIC, C_22_H_14_). Using steady-state spectroscopy and molecular modeling, we show that the charge-transfer (CT) character of the lowest electronic excitation can be continuously tuned by varying the fraction of spacer molecules, with the magnitude of the Davydov splitting (DS) serving as a convenient metric for the CT interaction^4,6^. From the modified CT interaction, the change in the singlet/triplet pair coupling matrix element that determines the SF rate can be computed^3,4^. Surprisingly, when measuring the SF rate using femtosecond transient absorption (TA) spectroscopy, we find that it is almost unchanged in going from a film containing only 15% PEN molecules to a 100% PEN film, despite substantial changes in the CT interactions as evidenced by the change in the absorption lineshape. This experimental observation is at odds with the theoretical prediction of an order-of-magnitude decrease of the SF rate and can be contrasted with previous reports of SF in PEN derivatives^18–20^. We propose that the robust nature of SF in these mixed films is due to a combination of mixing behavior and rapid singlet exciton diffusion to hot spots where SF is very fast. In blends exhibiting occupational disorder it appears that SF can be quite robust, even as the bulk exhibits large changes in intermolecular interactions.

## Results

### Structural and electronic properties

**Fig. 1:**
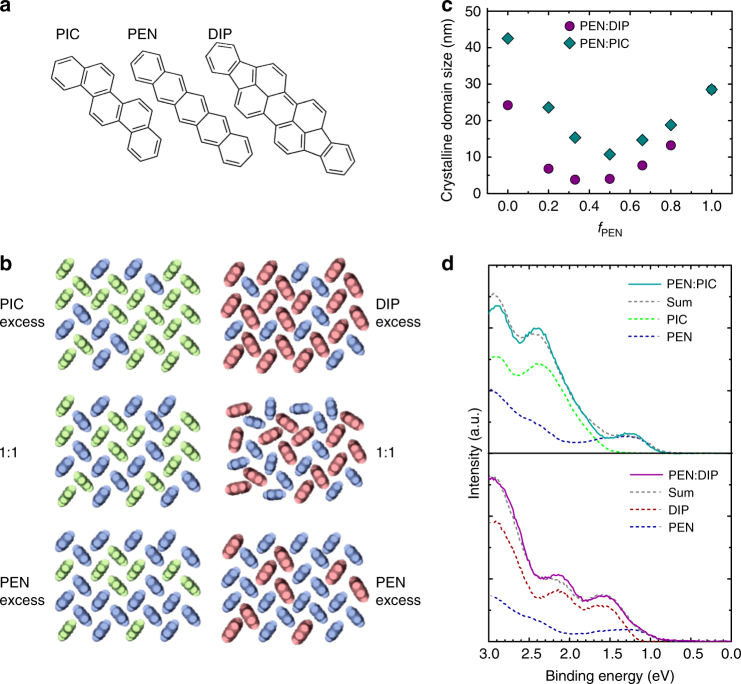
Structural and photoelectric characterization of the systems. **a** Chemical structure of PEN and the spacer molecules PIC and DIP. **b** Schematic mixing behavior (top view, parallel to the substrate surface) of PEN:PIC (left column) and PEN:DIP (right column). **c** Crystalline domain size extracted from grazing incidence X-ray diffraction^21,22^. **d** Ultraviolet photoelectron spectroscopy data of the HOMO region of PEN, DIP, PIC, and the respective equimolar blends. Binding energies are given relative to the Fermi level. The gray dotted lines are the sum of the HOMO regions of the pure compounds, weighed according to the mixing ratio

Before we discuss the effects of the spacer molecule on the intermolecular interactions and photophysics of pentacene, we briefly recapitulate the structural and electronic properties of the mixed films^21,22^. Both systems, PEN mixed with DIP or PIC (Fig. 1a), show a statistical mixing behavior (Fig. 1b) with a random occupation of lattice sites by either of the two components in a herringbone arrangement over a broad range of mixing ratios^21,22^. As the fraction of PEN molecules (*f*_PEN_) decreases, the average number of PEN molecules as nearest neighbors of a given reference PEN molecule decreases and the average distance between neighboring PEN molecules increases (Supplementary Fig. 1 and Supplementary Note 1). However, while the average fraction of PEN to spacer molecules is given by the mixing ratio, the random occupation of lattice sites leads to a distribution of molecular environments of a given PEN molecule (PEN or spacer) and intermolecular distances, which we will refer to in the following as occupational disorder. The mixed films are crystalline and exhibit orientational order, but the crystalline domain size (Fig. 1c) in the mixed films is reduced compared to films of the pure components by a factor of 2.5 (10) for PEN:PIC (PEN:DIP).

To demonstrate that the spacer molecules (DIP and PIC) do not interact strongly with PEN in the ground state we show ultraviolet photoelectron spectroscopy data of the pure compounds and the 1:1 blends (Fig. 1d). Data of the pure compounds are consistent with earlier results^23–25^. The density of states near the HOMO region in both mixtures can be described as the sum of the pure compounds, clearly showing their lack of interaction. This is in contrast to more strongly interacting mixtures that exhibit a partial or integer charge transfer or a hybridization of molecular orbitals in the ground state^26^. Note that small changes in the HOMO level peak shape in the mixtures compared to the pure compounds are consistent with the reduction of long range order shown in Fig. 1c ^27^.

### Steady-state optical spectra

**Fig. 2:**
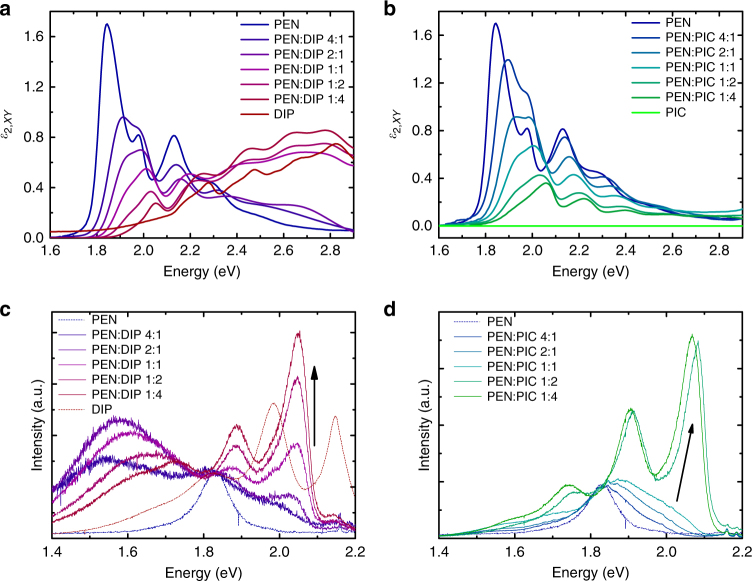
Optical properties of the systems. **a**, **b** Absorption spectra. Data of PEN:DIP blends are adapted from ref. ^28^. **c**, **d** PL-spectra. The intensities in **c** and **d** have been normalized to the intensity at 1.82 eV (PL-maximum of pure PEN). The arrows indicate changes with decreasing *f*_PEN_. For comparison, the pure film spectra are included with the exception of PIC, which does not show PL in the relevant energy range^29^

The absolute value of the DS in PEN is a measure of the CT character of the lowest electronic excitation^3,4^ and can be extracted from the absorption spectra of both blends (Fig. 2a, b and Supplementary Fig. 2). As *f*_PEN_ decreases, the shape of the spectra *ε*_2,xy_ (*E*) changes continuously. Apart from an overall decrease of the intensity, the relative intensity of the Davydov components at 1.85 and 1.98 eV changes rapidly, the absolute value of the DS decreases with decreasing *f*_PEN_ and the spectra shift toward higher energies. For high fractions of the spacer molecule, the spectra of both mixed systems are similar to the solution spectrum of PEN. This is most evident for the PEN:PIC blends in Fig. 2b since the pure PIC absorption spectrum does not overlap with the monomer PEN spectrum, whereas DIP absorption contributes to the spectra of PEN:DIP (Fig. 2a). The shift toward higher photon energies is caused by two effects: first, a continuous change in the polarizability of the local environment of a given PEN molecule due to the statistical intermixing^28^ and, second, a change in the electronic coupling between PEN molecules^3,4^. The second effect also results in the change in the relative intensities of the two Davydov components around 1.85 and 2.0 eV^4,5^.

Changing *f*_PEN_ also affects the photoluminescence (PL) spectrum of the mixed films (Fig. 2c, d) and, as *f*_PEN_ decreases, the spectral shape approaches the shape of the PL spectrum of monomeric PEN. This can be understood in terms of the increasing PIC and DIP concentrations leading to an increasing fraction of isolated PEN molecules, for which the probability of radiative relaxation is high, see also Supplementary Fig. 3 and Supplementary Note 2.

While we can exclude any contribution from neat PIC to the shape of the PL-spectra of the PEN:PIC-blends due to the different range in which PIC emits^29^, this is not obvious in the case of blends of PEN and DIP. For thin films of neat DIP, emission originates from low-energy states, most likely excimer species^30^. However, these species seem unaffected by the presence of PEN molecules as judged by the shape of the PL-spectra. For all mixing ratios, the total luminescence spectrum is a sum of the pure DIP emission plus the monomeric PEN emission, giving no evidence of complexation or exciplex formation between the PEN and the spacer molecules. This is further supported by fluorescence excitation spectroscopy measurements, see Supplementary Fig. 4. Increasing PIC and DIP concentrations simply leads to an increasing fraction of isolated PEN molecules, for which singlet fission cannot occur and that now can emit radiatively. However, the PL lifetime of the isolated PEN emission was <20 ps (the instrument response time for the streak camera) in all blends, suggesting that another loss mechanism is operative, most likely energy transfer to PEN aggregates as discussed below.

### Modeling of absorption spectra

Theory predicts that the DS should decrease as the separation between PEN molecules increases, mainly due to a decrease in the CT character of the lowest energy excitation^3,4^. In thin films of PEN mixed with DIP or PIC, the distance between neighboring PEN molecules increases gradually with increasing incorporation of DIP or PIC due to expansion of the lattice, and hence, the DS should decrease with decreasing *f*_PEN_. To confirm these qualitative expectations, we modeled two facets of the blended systems using a mixed Frenkel-CT Hamiltonian that incorporates vibronic coupling to one dominant intramolecular vibrational mode. We first modeled the effect of the lattice expansion on the DS by setting the Hamiltonian matrix elements that mix localized and charge-transfer electronic configurations (i.e., the charge-transfer integrals) to values calculated using the unit cell parameters of the mixed crystals at each *f*_PEN_. We also modeled the effect of occupational disorder on the DS by systematically replacing half the pentacene molecules with PIC or DIP and neglecting all pentacene–spacer interactions. Since our computational model assumes periodic boundary conditions, the replacement was done in such a way that preserved translational symmetry and maintained *f*_PEN_ = 0.5. In effect, this left us with two interleaved but non-interacting lattices; one lattice was composed entirely of pentacene molecules and the other of spacer molecules. Further details concerning the theoretical model are given in refs. ^3,4^ and Supplementary Note 3.

**Fig. 3:**
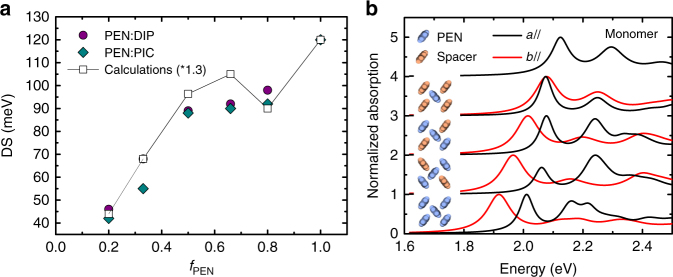
Effects of incorporation of spacer molecules on Davydov splitting. **a** Davydov splitting (DS) of the lowest electronic excitation in PEN in PEN:DIP (purple circles) and PEN:PIC (green diamonds). For comparison, the DS resulting from calculations based on the lattice parameters of PEN:DIP (white squares), which has been scaled by a factor of 1.3 in order to match the experimental results at *f*_PEN_ = 1, is also shown. **b** Comparison of the absorption spectrum of PEN if nearest neighbors (blue) are replaced by a spacer molecule (orange). In the mixed lattices *f*_PEN_ = 0.5 and periodicity is maintained. The components of the spectrum polarized along **a** and **b** are normalized by the absorption maximum

The results of the calculations concerning the effect of the expanded lattice on the DS, shown as white squares in Fig. 3a, display excellent agreement with the experimentally determined DS, shown as circles and diamonds for PEN:DIP and PEN:PIC, respectively. Even the plateau around *f*_PEN_ = 0.66 is captured qualitatively by our simulations, an effect related to the anisotropic changes in the charge-transfer integrals upon expansion of the unit cell (see e.g. ref. ^31^ for how changes in packing can affect the charge-transfer integrals). It has been shown previously that the DS in pure pentacene is driven almost entirely by charge-transfer interactions between nearest-neighbor molecules^4,32,33^. Coulombic (transition-dipole) interactions have only a small effect on the DS due to the weak, short-axis-polarized transition dipole moment. Hence, the observed changes in the DS with decreasing *f*_PEN_ can be ascribed mainly to changes in the electron and hole charge-transfer integrals between nearest-neighbor molecules as the lattice parameters change with *f*_PEN_. To further substantiate the attribution of the changes in the DS to changes in the charge-transfer integrals, consider a one-dimensional herringbone-like lattice in which only nearest-neighbor molecules interact via charge-transfer (i.e., no transition dipole coupling or coupling between non-nearest neighbor molecules). In such systems, the free exciton (no vibronic coupling) DS is equal to^3^

1 $${\mathrm {DS}} = \left| {\sqrt {2\left( {t_{\mathrm e} + t_{\mathrm h}} \right)^2 + 1/4\left( {E_{\mathrm {CT}}^2} \right)} - \sqrt {2(t_{\mathrm e} - t_{\mathrm h})^2 + 1/4\left({E_{\mathrm {CT}}^2} \right)}}\right|$$

where *t*_e_ and *t*_h_ are the electron and hole transfer integrals between nearest-neighbor (translationally inequivalent) molecules and *E*_CT_ is the energy of the CT state relative to a localized Frenkel excitation. In Eq. (1), the first and second terms correspond to the energies of the *k* = 0 symmetric and *k* = 0 antisymmetric excitons, respectively, which give rise to absorption polarized along the *b*-axis and perpendicular to it^3^. For a two-dimensional monoclinic herringbone lattice including only charge-transfer interactions between the four translationally inequivalent nearest-neighbor molecules the DS is similar to Eq. (1) except for the insertion of an additional factor of two in front of (*t*_e_ + *t*_h_)^2^ and (*t*_e_ −* t*_h_)^2^. Notably, the DS for the 2D lattice reduces to

2 $${\mathrm {DS}}= 2\left| {\left| {t_{\mathrm {e}} + t_{\mathrm {h}}} \right| - \left| {t_{\mathrm {e}} - t_{\mathrm {h}}} \right|} \right|$$

in the limit that the energy difference between localized Frenkel and CT states vanishes. Hence, the DS depends on the absolute and relative magnitudes of the CT integrals; as the magnitude of the integrals decreases the DS is also expected to decrease.

In triclinic pentacene, the four nearest-neighbor interactions are not all equal; as shown in ref. ^3^, the ±(1/2,1/2) and ±(1/2,−1/2) interactions are different. Although the corresponding DS is more complex than the simpler monoclinic example discussed so far, we can nevertheless use Eq. (2) to roughly estimate the free exciton DS of our pentacene mixtures after symmetrizing the four nearest-neighbor interactions so that a single set of (*t*_e_,*t*_h_) values apply to all four nearest-neighbor interactions, see Supplementary Tables 1 and 2. Supplementary Fig. 5 compares the DS calculated using Eq. (2) and the symmetrized values of *t*_e_ and *t*_h_ with the calculated DS from Fig. 3a. The DS predicted by Eq. (2) is systematically larger than that of the simulations presented in Fig. 3a, mainly due to the neglect of vibronic coupling^3^ and *E*_CT_; in pentacene, the value of *E*_CT_/2 (≈90 meV) needed to simulate the DS is much smaller than 2|*t*_e_+*t*_h_|, but is not negligible relative to 2|*t*_e_ − *t*_h_|^3^. This effect can be seen in the third data set in Supplementary Fig. 5, which is the DS calculated using using Eq. (1) with *E*_CT_/2 = 90 meV and an additional factor of 2 to make Eq. (1) applicable to two-dimensional lattices. Other smaller deviations between the calculated DS in Supplementary Fig. 5 and the DS from Fig. 3a arise from the neglect of charge–transfer interactions between nearest-neighbor equivalent molecules and Coulombic (i.e., dipole–dipole) coupling. Such effects are absent from Eq. (2) but are included in the simulations of Fig. 3a. To summarize, the similar trend between Eq. (2) and the calculated DS in Fig. 3a supports attributing changes in the DS as a function of *f*_PEN_ mainly to changes in the charge-transfer integrals between nearest-neighbor inequivalent molecules.

We also modeled the effect of occupational disorder on the DS by replacing PEN molecules with DIP and PIC spacer molecules. In these calculations the *t*_e_,*t*_h_ values for the pure PEN lattice^3^ were used for the PEN–PEN interactions, but the PEN–spacer interactions were taken to be zero. The absorption spectra corresponding to different substitution patterns are displayed in Fig. 3b. As expected, the strongest change is observed if all translationally inequivalent nearest neighbors surrounding a given pentacene molecule are replaced by spacer molecules, leading to the disappearance of the DS. Note, in this configuration *f*_PEN_ is 0.5, as each spacer molecule is also surrounded by four PEN molecules in the periodic lattice. In this case, the interactions between the equivalent pentacene molecules leads to a slight red shift compared to the monomer spectrum. Interestingly, when half of the nearest neighbors are spacer molecules the DS depends on whether the spacer molecules appear at the positions ±(1/2,1/2) or ±(1/2,−1/2); however, the average DS is not that different from the DS in the pure PEN lattice. To summarize, the important conclusion from the simulations contained in Fig. 3 is that most of the change in the DS originates from the expanded lattice due to the spacer molecules and that occupational disorder has a lesser effect on the observed DS.

### Experimental determination and modeling of singlet fission rates

The isolated PEN molecules that give rise to the PL spectra in Fig. 2c, d only represent a small fraction of the total, since the bulk of the PEN molecules that give rise to the steady-state absorption do not emit due to rapid SF. To probe the details of the photophysics of the samples and in particular the dynamics of the dark PEN molecules, we utilize femtosecond TA spectroscopy, a tool that has been used successfully before to measure SF dynamics in solid-state PEN systems^9,14,16,17^.

**Fig. 4:**
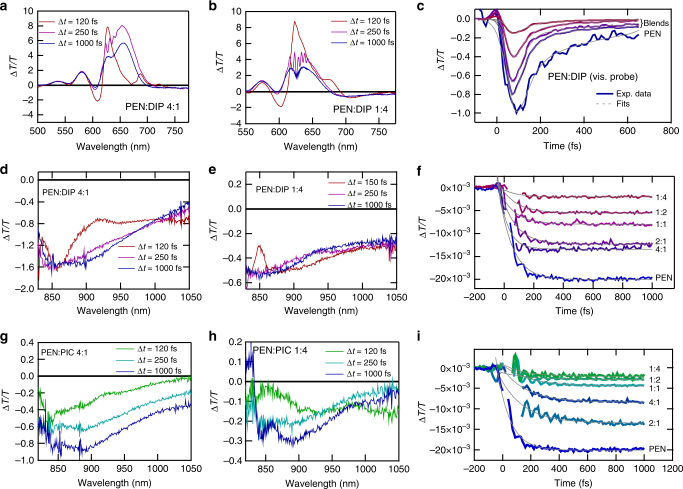
Photophysics of the blends. **a**–**f** TA spectra of two PEN:DIP blends with excitation at 620 nm and corresponding dynamics of all mixing ratios extracted at **c** 607 or **f** 875 nm. **a**–**c** visible probe, **d**–**f** near-infrared probe. **g**–**h** TA spectra of two PEN:PIC blends with excitation at 620 nm and near-infrared probe. **i** Corresponding dynamics of all PEN:PIC mixing ratios extracted at 875 nm

In the solid state, the PEN triplet displays a broad photoinduced absorption (PA) that extends across the near infrared, and the growth of this feature, along with the decay of the PA of the singlet, provides a straightforward way to extract the SF dynamics^17^. The pump center wavelength was fixed at 600–620 nm to selectively excite the PEN molecules, and avoid excitation of the DIP and PIC spacer molecules. Figure 4c shows the Δ*T*/*T* dynamics measured at the probe wavelength of 607 nm using 70 fs pump pulses centered at 620 nm. At this wavelength, the signal is a combination of ground-state bleaching (GSB) and singlet PA^17^. For all PEN:DIP blends, the singlet PA decays within 200 fs, leaving a combination of long-lived GSB overlapped with the tail of the triplet PA. These results already suggest that the SF in these blends occurs on the 100-fs timescale and is not strongly affected by the PEN concentration. To study the triplet population build-up dynamics, we used shorter, sub-20-fs pump and probe pulses and concentrated on the near-infrared spectral region, where the PA of the triplets in PEN peaks. Fig. 4f, i) show the rise of the triplet PA for different PIC and DIP blends, along with fits to the data. In all cases, the build-up of the triplet population was clearly resolved and was complete within 500 fs after excitation.

The surprising result from our ultrafast TA experiments is the substantial invariance in the SF rates, despite the large changes in the steady-state absorption and PL spectra, since the same CT interactions that vary the DS in the absorption spectrum are also expected to play a key role in SF^4,34^. To investigate this further, we modeled singlet fission in the mixed crystals using a similar model Hamiltonian as applied in the analysis of the DS, yet including a larger number of vibrational modes. The quantum dynamics calculations of SF have been carried out using the multi-configuration time-dependent Hartree (MCTDH) method^35^ in the linear vibronic coupling approximation. We solved a trimer model (with three pentacene molecules along the herringbone direction) accounting for 9 electronic states and 66 intramolecular vibrational modes.

The diagonal terms of the Hamiltonian read:

3 $$h_I\left( {\boldsymbol{x}} \right) = \mathop {\sum }\limits_i \frac{{\omega _i}}{2}\left( {x_i^2 + p_i^2} \right) + \mathop {\sum }\limits_i k_i^Ix_i + E_I$$

where the first term expresses harmonic oscillators and *E*_*I*_ is the diagonal state energy. *k*_*i*_^*I*^ denote the intra-molecular vibronic couplings to vibrational modes *i* (i.e., spectral density), which are taken from an earlier study^36^. The intra-molecular atomic displacements from the ground state to the triplet, cation, anion, and S_1_ are calculated based on the optimized geometries of monomer by DFT with the PBE functional. The frequency, *ω*_*i*_, and the displacement vectors of normal modes are also calculated by DFT at the ground-state geometry. Then, the Cartesian atomic displacements are projected on the normal modes and the vibronic couplings *k*_*i*_^*I*^ are extracted.

**Fig. 5:**
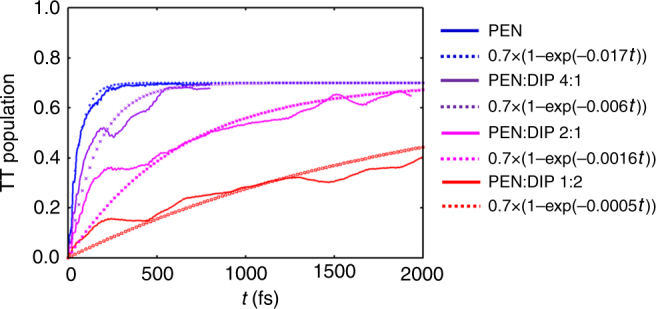
Calculated evolution of triplet pair population. Population of triplet pair (TT) from quantum dynamics calculations (solid lines) and exponential fits (dotted lines). At *t* = 0, a singlet exciton is localized on the center molecule of the herringbone trimer model

Figure 5 shows the time evolution of the TT populations obtained from the quantum dynamics calculations together with exponential fits. As the DIP ratio in the crystal increases, the intermolecular couplings mixing Frenkel and CT excitations decrease, and so does the SF rate.

**Fig. 6:**
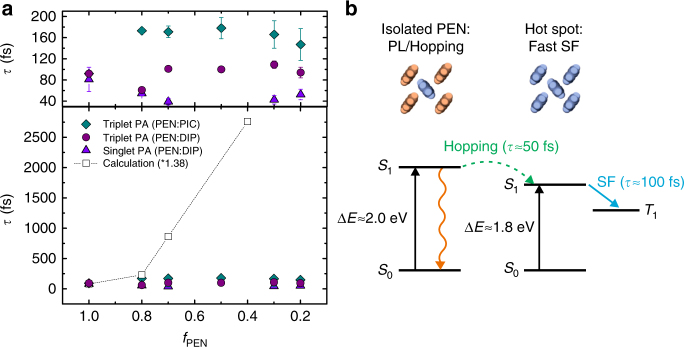
Singlet fission time constants and proposed model. **a** Comparison of experimental results (decay times of the singlet PA extracted at 607 nm in PEN:DIP and build-up times of the triplet PA extracted at 875 nm in PEN:DIP and PEN:PIC, respectively) with the calculated SF rate based on the model discussed. The upper panel shows the experimental time constants in the lower panel with an expanded scale. The error bars indicate the uncertainty of the fit. The results of the calculations have been scaled by 1.38 to match the experimentally determined SF time constant of pure PEN. Deviations between model and experiment are discussed in the text. **b** A schematic sketch of the potential origin of the deviations of the experimental results from the theoretical predictions showing the two extreme cases of PEN with and without PEN nearest neighbors

In Fig. 6, we compare the measured SF time constants in the PIC and DIP blends with those predicted by the theory described above. The experimental and theoretical SF times are scaled to match at *f*_PEN_ = 1.0.

## Discussion

Clearly there is a discrepancy between the model and the experiment. The model predicts an increase of the SF time constant by more than a factor of 30 from 80 to 2800 fs as *f*_PEN_ decreases to 0.4, while the experimental rates show no notable change over the same range of concentrations. The origin of this discrepancy likely does not lie in the experiments, which were reproducible using samples prepared at different times, as well as using different laser spectroscopy systems. The fact that two chemically distinct spacer molecules give essentially the same result shows that this is a general phenomenon that does not arise from specific molecular interactions between PEN and the spacer molecules. Since the physical model linking the SF rate to the CT interaction has been used successfully to model SF in structurally well-defined systems like crystalline films and dimers^10,12,20^ it is more likely that the insensitivity of the SF time constant to *f*_PEN_ results from the random occupation of lattice sites in these mixed films and the corresponding distribution of lattice spacing and chemical nature of nearest neighbors (PEN or spacer). While, at low PEN fractions, the majority of the PEN molecules have spacer molecules as nearest neighbors, there will always be PEN molecules with other PEN molecules as nearest neighbors due to the statistical intermixing. Supplementary Table 3 summarizes the probability for nearest-neighbor configurations at different mixing ratios. These regions of the film can act as fission hot spots where the exciton can undergo rapid SF assuming it can reach them. In the blended films, the excitons created on PEN molecules with at least one spacer molecule as a nearest neighbor have higher energies than those at lattice sites with only PEN neighbors. With a downhill energy transfer pathway, excitons created in these regions will quickly diffuse to the lower energy PEN aggregates and undergo rapid SF. The singlet exciton diffusion rate in PEN has not been directly measured, but if we assume it is similar to that of tetracene, we can estimate a hopping time of 50 fs^37^ even in the absence of a downhill energy path. This is rapid enough to allow several hops that can transport an exciton from a slow fission region to a fast fission region. An estimation of the size of these hot spots is difficult, but based on the X-ray diffraction data we can estimate an upper limit for the crystalline domain size, or a maximum cluster size, in the equimolar PEN:DIP blend of 4 nm, corresponding to a few tens of PEN molecules. A schematic sketch summarizing our hypothesis is shown in Fig. 6 for the two extreme cases of PEN with and without PEN nearest neighbors.

The dramatic effects of varying *f*_PEN_ on the steady-state absorption and PL spectra are not accompanied by changes in the dynamics because these stationary ensemble measurements are relatively insensitive to local inhomogeneities like hot spots. Dynamic measurements like TA spectroscopy, on the other hand, can be very sensitive to small concentrations of structural inhomogeneities due to facile exciton transfer. To quantitatively simulate the data using the mechanism proposed would require more detailed information on energy transfer rates, molecular distributions, and packing variations, but such a study is beyond the scope of the present work.

While dilution with PIC or DIP has little effect on the SF rate, there is a significant decrease in the triplet relaxation rate of the PEN component as *f*_PEN_ decreases (Supplementary Fig. 7). While the long-time dynamics of the triplets is not the focus of this work, this observation deserves some discussion. In PEN, there are two triplet loss mechanisms: a bimolecular triplet–triplet annihilation (TTA) pathway that leaves one surviving triplet, as well as an internal conversion back to the ground state that is relatively rapid in polycrystalline films^38,39^. In our mixed samples, both pathways can be modified: geminate TTA could be enhanced due to local caging of the triplet pair (although this is less likely in domains with a few tens of PEN molecules than in covalent dimers^19,40,41^), while nongeminate TTA could be suppressed due to slower long-range triplet diffusion. Meanwhile, internal conversion is expected to slow down as *f*_PEN_ decreases and the PEN molecules see more inert molecules instead of each other. We tentatively assign the longer triplet lifetime to slower internal conversion in the mixed films, but reliably distinguishing between these different possibilities requires a detailed study of the fluence dependence of the triplet dynamics on nanosecond timescales.

The robust nature of singlet fission with respect to occupational disorder has been inferred previously from the fact that it can occur rapidly even in solution^42^, and these previous results have also been explained in terms of rapid singlet exciton diffusion to preferred SF sites. In thin films, diffusion to low-abundance defects has a strong impact on SF^15,43^ since sites, where the local symmetry is broken, can act has hot spots favorable for SF^44^. Theoretical studies of singlet fission in pentacene have highlighted the co-existence of multiple CT-mediated pathways that cancel out in the single crystal at 0 K^4,45^. Such destructive interference effects are, however, washed away in the presence of static or (thermal) dynamic effects^46^. SF hot spots could thus correspond to local domains where symmetry constraints are most efficiently released, hence leading to the fastest singlet fission events. We note that such SF hot spots have been proposed as the origin of efficient singlet fission in amorphous films of 5,12-diphenyl-tetracene^15^. The important difference to our samples is the lack of a preferred orientation of the molecules, which leads to a large variety of molecular packing geometries and accordingly, complicated kinetics involving multiple fission rates. The fact that we observe only one fast fission rate suggests that the fission hot spots coincide with the lowest energy sites. Interesting in this context is a recent publication on amorphous films of rubrene mixed with spacer molecules^47^, in which a continuous change of SF rate is observed using time-resolved fluorescence spectroscopy. This contrasts with our results as it seems that diffusion to defect sites does not notably impact the kinetics and might point towards a complex relation of orientational order, energy transfer, and singlet fission in such diluted systems. We note here that we observe a dependence of the SF rate on the fraction of PEN molecules, if DIP is used as a spacer molecule and 400 nm is chosen as excitation wavelength (Supplementary Fig. 8). In this case, DIP is excited and transfers the excitation energy to the *S*_1_ state of PEN, leading to a significant change in the SF time constants.

Finally, the role of defects as hot spots for SF in samples with long-range order has been demonstrated before by comparing single crystals and polycrystalline films of tetracene^43^. The increase of the SF rate in polycrystalline films has been explained by singlet exciton diffusion to defect sites where the molecular arrangement is favorable for SF, however, without relating the results to intermolecular interactions governing the SF. The remarkable aspect of the results in this paper is that we can continuously tune the electronic CT interaction in the bulk film that is believed to control the SF rate, yet the experimental rate remains almost unaffected. We believe that our results have important implications for future studies of structure–property relations, as they demonstrate that small fluctuations in local molecular arrangement in films exhibiting long-range order can lead to the formation of hot spots that completely dominate the kinetics. In summary, we have demonstrated a method for the tuning of bulk intermolecular interactions in PEN over a large concentration range by mixing with weakly interacting spacer molecules, namely PIC and DIP. The changes in the absolute values of the DS could be simulated by a reduction of the charge transfer character of the lowest energy excitation caused mainly by an increase in the lattice spacing. The most remarkable result of this work is that these dramatic changes in the bulk electronic states have only a small effect on the experimental SF rate. In contrast, a theoretical model that quantitatively reproduces the changes in steady-state spectra and in the DS predicts a slowing down of SF over several orders of magnitude. We hypothesize that SF is robust in the face of such large-scale changes in intermolecular interactions due to a combination of rapid singlet exciton diffusion and the presence of localized fission hot spots in the film. This work emphasizes the necessity of taking occupational disorder and low-abundance structural features into account when analyzing exciton dynamics. In particular a modeling of SF rates based on single-crystal packing geometries might not always be sufficient.

## Methods

### Sample preparation

Mixed films of PEN (purchased from Sigma Aldrich, 99.9% purity) and PIC (purchased from NARD Co. with 99.9% purity) or DIP (Institut für PAH Forschung Greifenberg, 99.9% purity) were grown by organic molecular beam deposition at a base pressure of 2 × 10^−10^ mbar on Si substrates covered with a native oxide layer and on fused silica substrates at 297 K substrate temperature^22,28^. For both mixed systems samples with five molecular mixing ratios (4:1, 2:1, 1:1, 1:2, and 1:4) were prepared. The mixing ratio was determined before growth using a quartz crystal microbalance (QCM) calibrated using X-ray reflectivity, which was also used to monitor the deposition rate of 0.3 nm/min (PEN:PIC) or 0.25 nm/min (PEN:DIP). The nominal film thickness was 20 nm, corresponding to ~13 monolayers.

### Optical characterization and UPS

The absorption spectra were investigated during growth using differential reflectance spectroscopy (DRS), probing the in-plane component (*ε*_2,xy_ (*E*)) of the dielectric function on the fused silica samples. The DRS setup contained a fiber-coupled USB2000 spectrometer and a DH-2000 lamp (Ocean Optics) covering the energy range 1.5–2.9 eV. *ε*_2,*xy*_ (*E*) was extracted by a Kramers–Kronig constrained variational analysis approach^48–51^ for PEN:PIC blends. Since PIC absorbs strongly above the measured energy range, absorption above 2.9 eV was taken into account by a parametrized extrapolation of *ε*_2,*xy*_ (*E*). Photoluminescence (PL) spectroscopy was performed post growth on the Si samples using a Horiba Jobin Yvon LabRAM HR spectrometer under N_2_ atmosphere using a wavelength of 532 nm for excitation. Ultraviolet photoelectron spectroscopy with He I was performed using a home-built UHV system equipped with an Omicron EA125 HR hemispherical analyzer in angular integration mode. Steady-state excitation spectra were acquired on a Horiba Fluorolog-3 DF spectrofluorimeter. The excitation light source was a 450 W continuous xenon lamp, emission was monitored at 90° using a Hamamatsu R2658P PMT.

### Transient absorption spectroscopy

The pump-probe set-up^52^ consisted of an amplified laser system (Libra, Coherent), which delivers a 1 kHz pulse train of 4 mJ, 100 fs pulses at 800 nm. A portion of the beam of 180 μJ energy was used to pump an optical parametric amplifier (OPA) seeded by a white light continuum generated in a 2 mm Sapphire plate, which was spectrally filtered (10 nm FWHM, centered at 620 nm) and amplified in a 1-mm-thick BBO crystal by the second harmonic of part of the fundamental beam. The probe pulse was a white light continuum generated by focusing a small fraction of the 800 nm beam in a 2 mm Sapphire plate. The instrument response function (IRF) of the system was about 80 fs, largely limited by the pump pulse duration. Pump and probe beams were synchronized by a delay line and focused on the sample by a lens with a focal length of 500 mm and a spherical mirror with 200 mm focal length, respectively, assuring that the pump spot size was larger than the probe spot size. The pump beam was blocked after the sample by an iris. The differential transmittance signal (Δ*T*/*T*) was obtained by dispersing the probe pulse on an optical multichannel analyzer (OMA) and by subtracting pump-on and pump-off spectra. In a different set of experiments, two synchronized non-collinear OPAs were used to generate sub-20-fs pulses in the visible (centered at 620 nm) and in the IR (centered at 875 nm), used as pump and probe pulses, respectively.

### Data availability

Experimental data are available from the corresponding author on request. For details on theoretical models, see author contributions.

## Electronic supplementary material

Supplementary Information
