# Supplementary material for: Robust singlet fission in pentacene thin films with tuned charge transfer interactions
Source: Nat Commun. 2018 Mar 5;9:954. doi: 10.1038/s41467-018-03300-1 (PMC5838205; doi:10.1038/s41467-018-03300-1)
Supplement: Supplementary file 1 — Supplementary Information [file 41467_2018_3300_MOESM1_ESM.pdf]

Supplementary Information:
“Robust singlet fission in pentacene thin films with tuned charge transfer interactions”

K. Broch et al.

Supplementary Figures

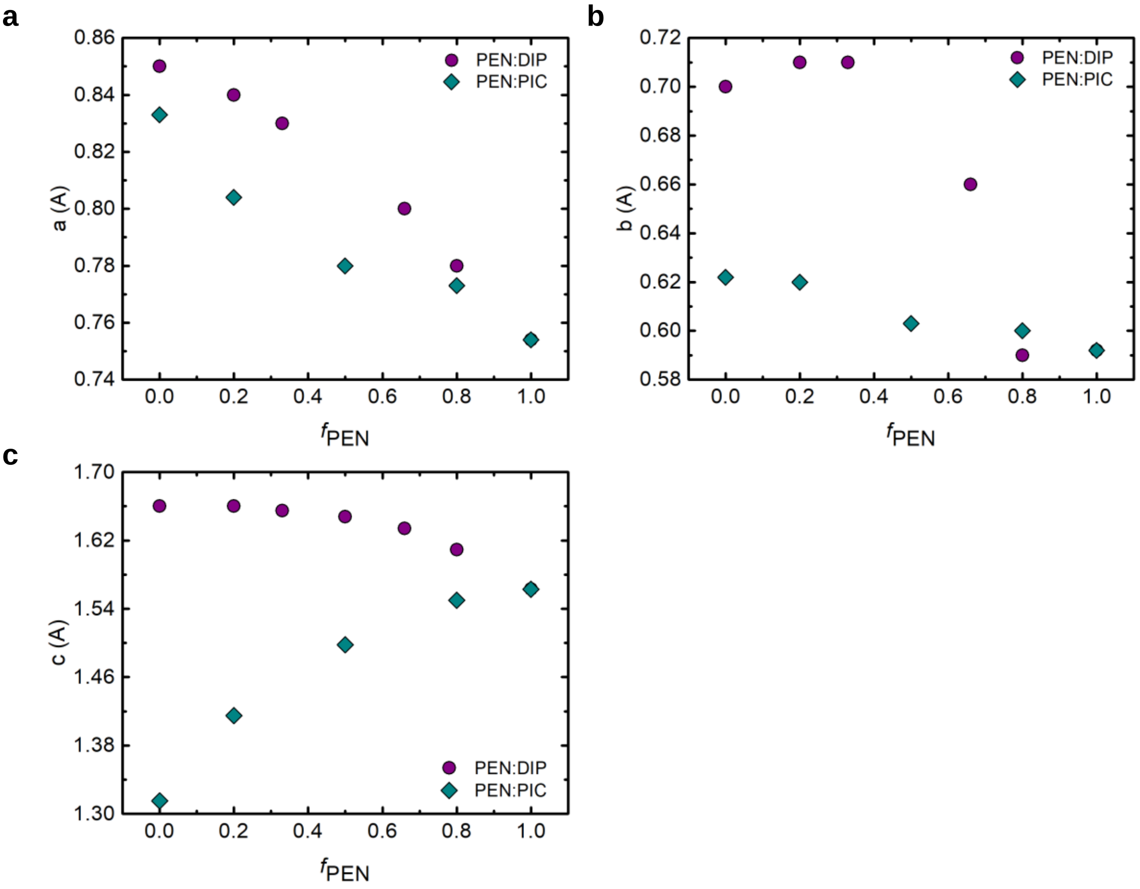

Supplementary Figure 1| Structure of PEN thin films mixed with spacer molecules.
a) - c) Change in lattice constants as the PEN-fraction (f_{PEN}) decreases for PEN:DIP (purple circles) and PEN:PIC (green diamonds). Data based on Supplementary References [1, 2].

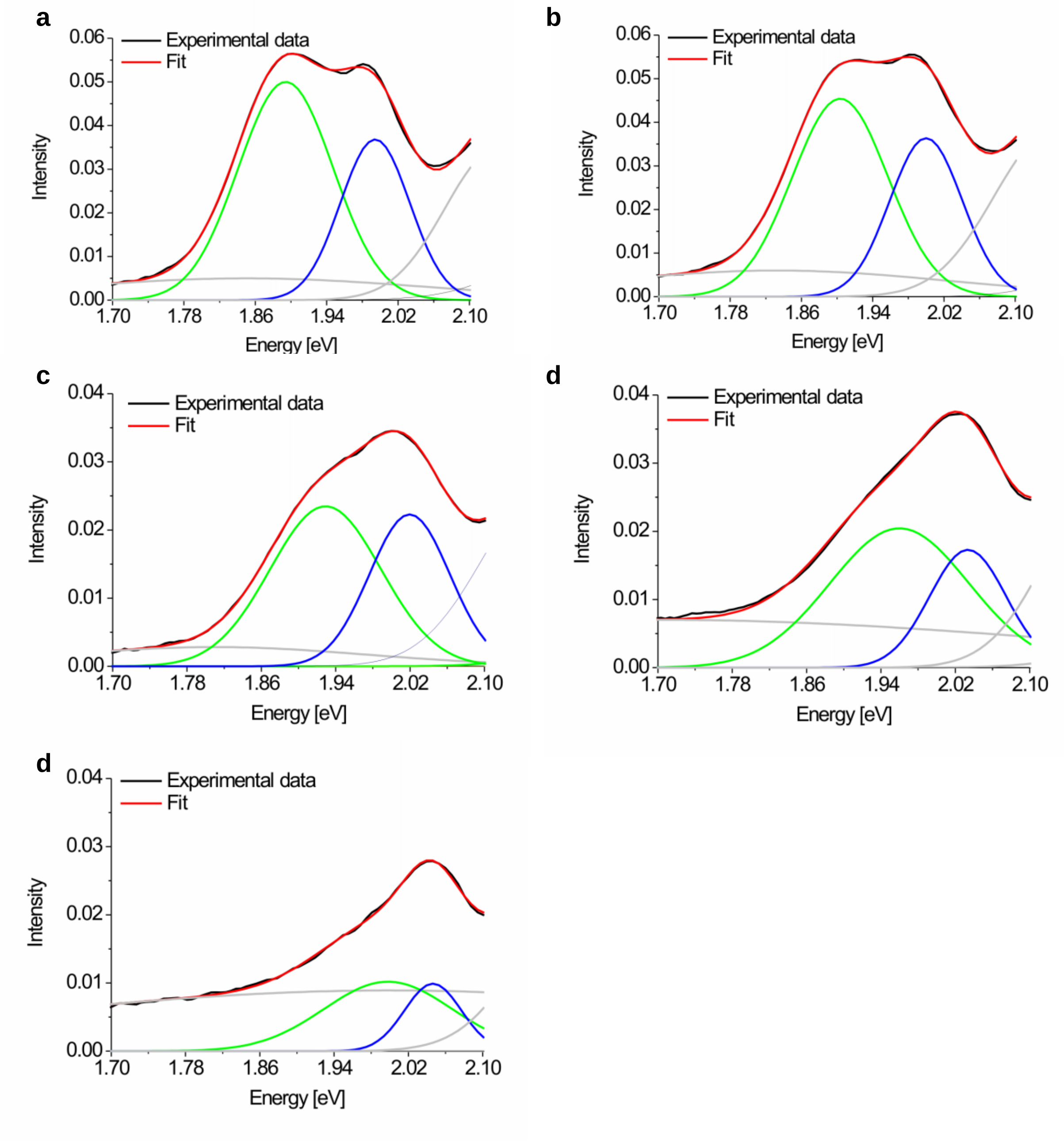

Supplementary Figure 2| Fits of the absorption spectra. Mixing ratio PEN:DIP a) 4:1, b) 2:1, c) 1:1, d) 1:2 and e) 1:4. The absorption spectra have been fitted with Gaussian oscillators to determine the Davydov-splitting.

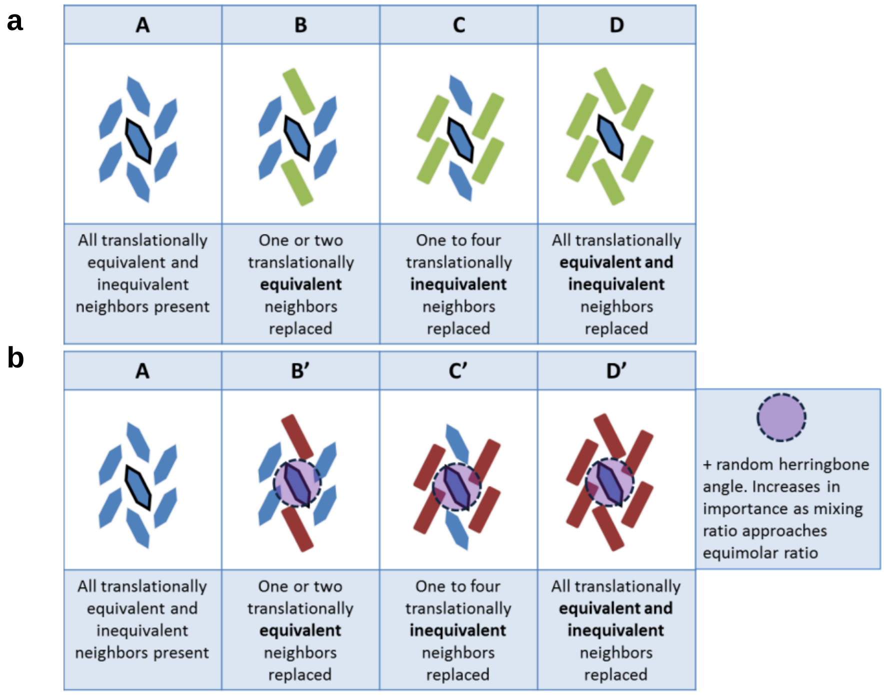

Supplementary Figure 3| Simplified schematic sketches of the local molecular environment of different reference PEN molecules in the blends. a) PEN:PIC, for which the herringbone angle is well-defined for all mixing ratios. b) PEN:DIP, for which there is potentially a random herringbone angle due to the decrease in long-range order parallel to the substrate surface.

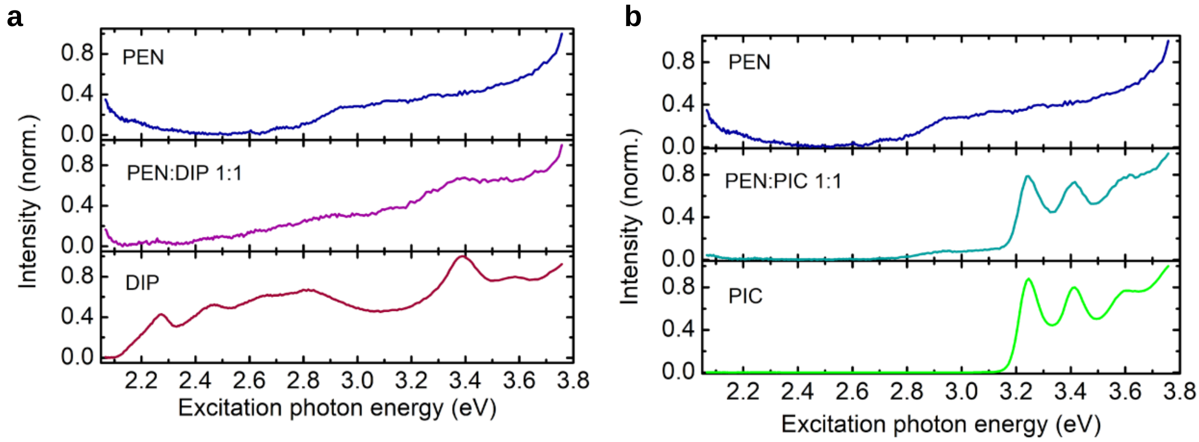

Supplementary Figure 4| Fluorescence excitation spectra. a) PEN:DIP and b) PEN:PIC. In both cases we probed the emission at a wavelength of 620nm. In both systems we observe energy transfer from the spacer molecule to PEN. For PIC as spacer molecule, this energy transfer is only relevant above 3.1eV (400nm) and thus, is not impacting our results. For DIP as spacer molecule, we observe energy transfer if an excitation energy >2.2eV (560nm) is chosen. Importantly, we find neither for DIP nor for PIC as spacer molecule any indication for complexation or excimer formation with PEN.

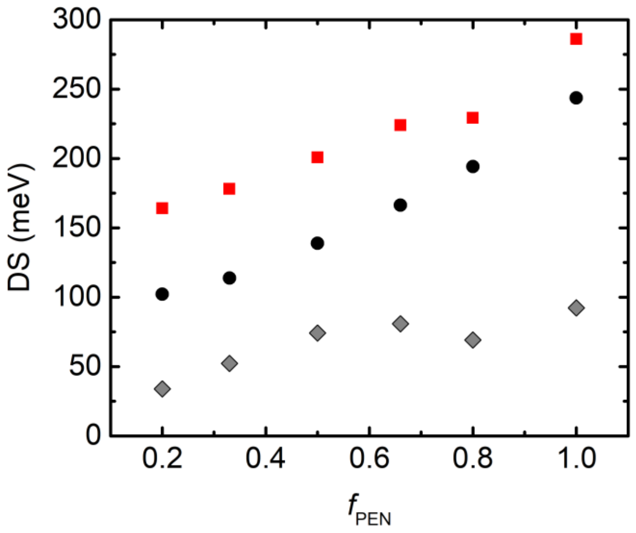

Supplementary Figure 5| Comparison of theoretical results for the DS. The DS calculated from equation (2) (red) is compared with the DS calculated from equation (1) (black), with $E_{CT}/2$ = 90 meV, and the unscaled DS values calculated numerically from Figure 3a (dark grey). To make equation (1) (black) applicable to two-dimensional lattices, a factor of 2 was included as described in the main text. In addition, the t_e and t_h values used in Eq.(1) and (2) were taken from Table S2. Although the DS predicted by equation (2) are much higher than the corresponding numerical calculations (due mainly to the neglect of vibronic coupling), the overall trend is captured. Hence, changes in the DS with changing f_{PEN} can be attributed mainly to changes in the charge-transfer integrals between inequivalent nearest-neighbor molecules as the unit cell expands with incorporation of either PIC or DIP.

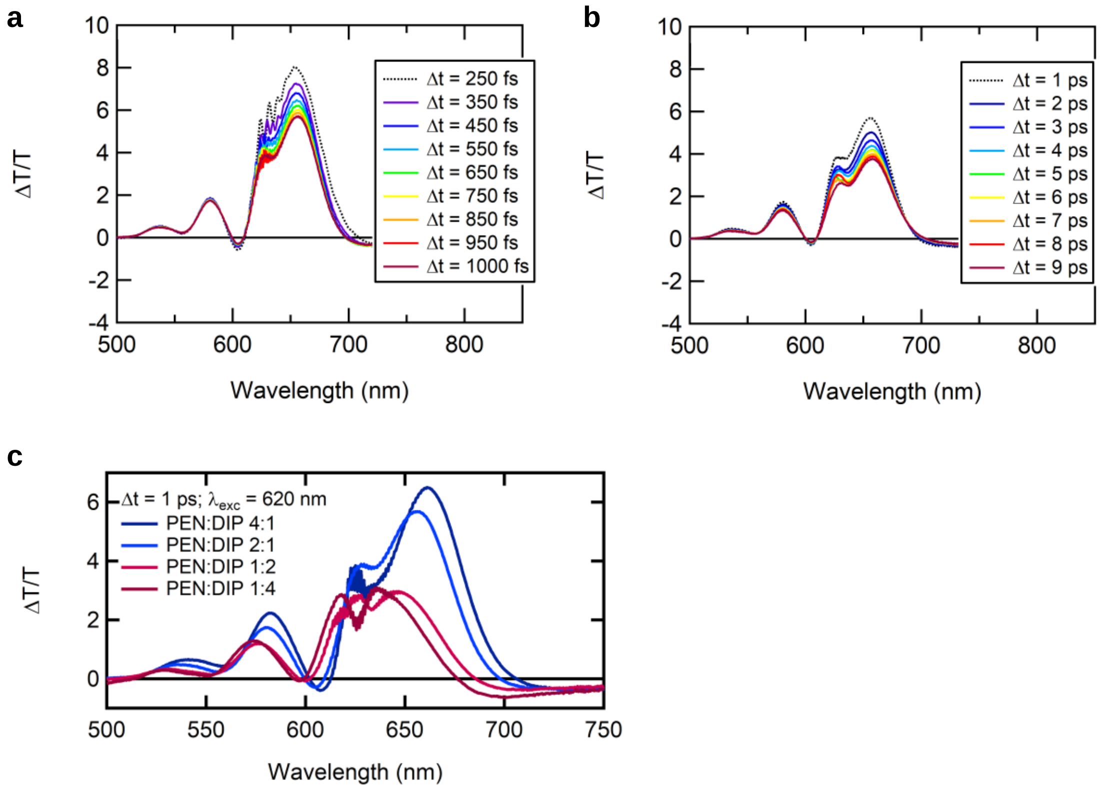

Supplementary Figure 6| Transient absorption spectra for longer delays. a), b) Transient absorption spectra of PEN:DIP 2:1 with 70fs pulses for delays longer than the 250fs shown in Fig. 4 in the main text. The pump center wavelength was fixed at 600-620 nm to avoid excitation of the DIP and PIC spacer molecules and to observe only the PEN dynamics. c) Comparison of the shape of the TA-spectra for different mixing ratios of PEN and DIP at a delay of 1ps. The fact that the spectral shape is not changing significantly for the two blends with more DIP and that a clear Davydov splitting is observed, might indicate that the dynamics is dominated by hot spots, which facilitate CT interactions between PEN molecules.

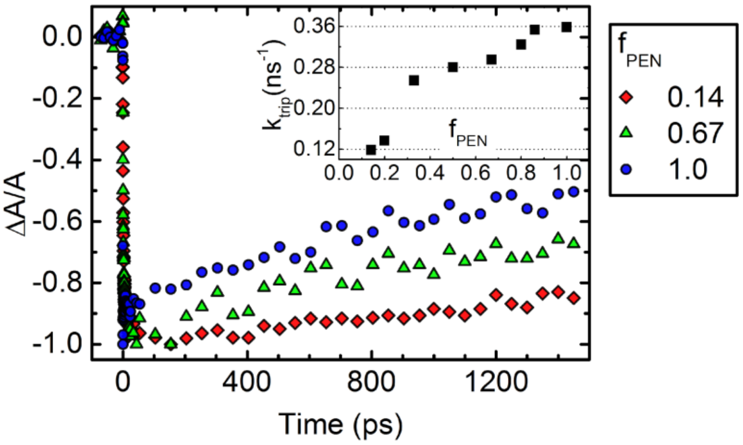

Supplementary Figure 7| Triplet dynamics. Ground state bleach and $T_1 \rightarrow T_n$ absorption in blends of PEN:DIP after excitation at 400nm. Long-time decay of the ground state bleach signals for f_{PEN} = 1.0 (blue circles), 0.67 (green triangles) and 0.14 (red diamonds). Dilution of PEN by DIP molecules extends the triplet lifetime. After 200 ps, both the GSB and the $T_1 \rightarrow T_n$ absorption in the NIR decay over the next 1.5ns. These decays, which are plotted parallel to each other, can be attributed to relaxation of the triplets back to the ground state. Inset: k_{trip} for different f_{PEN} as determined from fitting the bleach decays over the 1.5 ns time range showing the increase in the decay rate of the triplet by a factor of 3 as f_{PEN} is increasing.

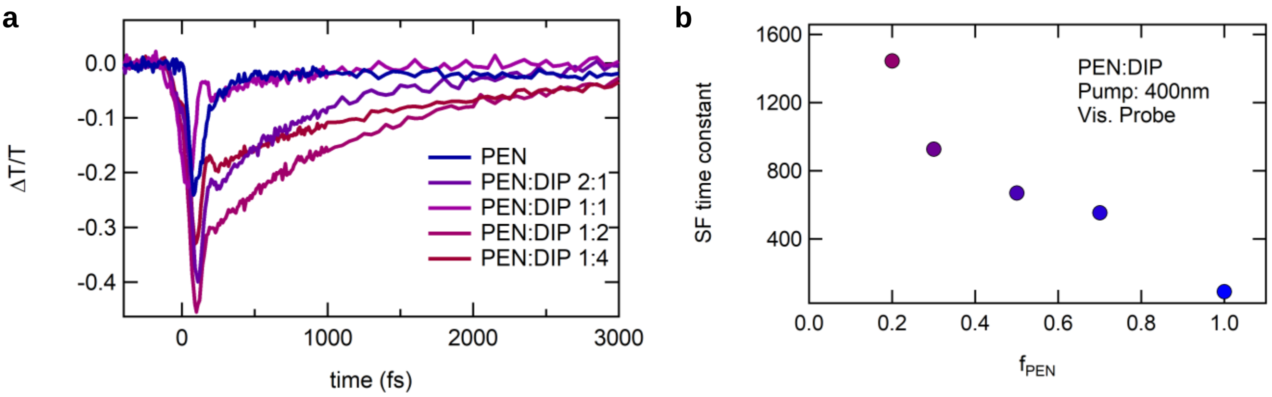

Supplementary Figure 8| Effect of energy transfer. a) Dynamics and b) SF time constant extracted at 610nm in PEN:DIP blends with an excitation wavelength of 400nm (3.1eV), where both, DIP and PEN, absorb.

Supplementary Tables

f_{PEN}		t_h (meV)	t_e (meV)
0.2	(0,1)	-13.8	-16.4
	(1,0)	-0.197	-0.455
	(1/2, -1/2)	33.9	44.0
	(1/2, 1/2)	-48.1	-44.2
0.33	(0,1)	-13.8	-16.4
	(1,0)	-0.225	-0.516
	(1/2, -1/2)	36.7	46.8
	(1/2, 1/2)	-52.3	-47.1
0.5	(0,1)	-18.6	-22.6
	(1,0)	-0.251	-0.669
	(1/2, -1/2)	40.5	55.5
	(1/2, 1/2)	-59.9	-55.0
0.66	(0,1)	-25.4	-31.5
	(1,0)	-0.245	-0.822
	(1/2, -1/2)	43.8	65.6
	(1/2, 1/2)	-68.1	-64.2
0.8	(0,1)	-53.6	-72.9
	(1,0)	-0.319	-1.11
	(1/2, -1/2)	40.8	88.7
	(1/2, 1/2)	-73.7	-84.6
1.0	(0,1)	-48.8	-65.2
	(1,0)	-0.414	-1.48
	(1/2, -1/2)	50.8	96.3
	(1/2, 1/2)	-92.2	-94.0

Supplementary Table 1| Calculated charge-transfer integrals for pentacene in a PEN:DIP blend as a function of f_{PEN}. The nearest-neighbor charge-transfer integral values (t_e,t_h) for the (0,1), (1,0), (1/2,1/2) and (1/2,-1/2) interactions evaluated using the methods presented **in Supp Note 3.**. These (t_e,t_h) values have been used for the calculations shown in Fig. 3 and pertain to the triclinic pentacene unit cell with unit cell parameters corresponding to the PEN:DIP blends shown in Supplementary Fig. 1.

f_{PEN}	t_h (meV)	t_e (meV)
0.2	41	44.1
0.33	44.5	47.0
0.5	50.2	55.3
0.66	56.0	64.9
0.8	57.3	86.6
1.00	71.5	95.2

Supplementary Table 2| The mean nearest-neighbor charge-transfer integrals for pentacene as a function of f_{PEN}. In triclinic pentacene the (t_e,t_h) values for the (1/2,1/2) and (1/2,-1/2) interactions are slightly different, see Table 1 and Supplementary Reference 3. To use Eq.'s (1) and (2) for monoclinic lattices in which the (1/2,1/2) and (1/2,-1/2) interactions are equal, mean values of te and th were obtained by averaging together the two nearest-neighbor |t_e | values and the two nearest-neighbor |t_h| values in Supplementary Table 1.

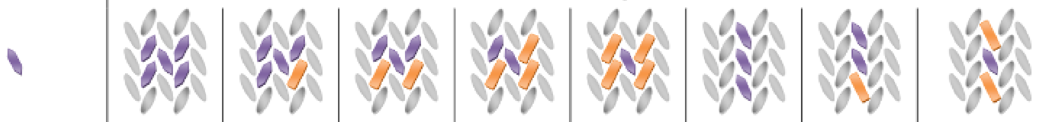

Fraction molecule A	Probability for							
	4	3	2	1	0	2	1	0
	translationally inequivalent A-neighbors					translationally equivalent A-neighbors		
1.00	1.000	0.000	0.000	0.000	0.000	1.000	0.000	0.000
0.80	0.410	0.410	0.154	0.026	0.002	0.640	0.320	0.040
0.75	0.316	0.422	0.211	0.047	0.004	0.563	0.375	0.063
0.67	0.202	0.397	0.293	0.096	0.012	0.449	0.442	0.109
0.50	0.063	0.250	0.375	0.250	0.063	0.250	0.500	0.250
0.33	0.012	0.096	0.293	0.397	0.202	0.109	0.442	0.449
0.25	0.004	0.047	0.211	0.422	0.316	0.063	0.375	0.563
0.20	0.002	0.026	0.154	0.410	0.410	0.040	0.320	0.640

molecule B   undefined A or B

Supplementary Table 3| Probability of nearest neighbour configurations. Probabilities shown for different fractions of molecule A (PEN) in blends with molecule B (DIP or PIC) in case of random occupation of lattice sites. The sketches show the nearest neighbour configuration along the molecular long axis corresponding to a top view of a thin film.

Supplementary Notes

Supplementary Note 1

Supplementary Figure 1 shows a comparison of the changes in the unit cell parameters depending on the mixing ratio (molar fraction of PEN, f_{PEN}) based on Refs. [21] and [22]. In both systems, Bragg-peaks originating from pure domains of PEN, DIP or PIC could not be observed for most mixing ratios, indicating complete intermixing of PEN and the respective spacer molecule (PIC or DIP), Supplementary References [1, 2]. The exception are blends with a large excess of PIC-molecules, for which the formation of phase separated domains of pure PIC-crystallites has been observed Supplementary Reference [2].

As the fraction of PEN molecules (f_{PEN}) decreases, the unit cell parameters change continuously from values close to PEN thin films to values close to the respective spacer molecule. This indicates statistical mixing with a random occupation of lattice sites by either of the two components in a herringbone arrangement Supplementary References [1, 2]. Furthermore, the average distance between neighboring PEN molecules increases with decreasing f_{PEN}, Supplementary References [1, 2]. The change in the unit cell parameters a and b is comparable in PEN:DIP and PEN:PIC blends, but interestingly, the change in the out-of-plane lattice spacing is opposed in PEN:DIP and PEN:PIC, which is caused by the length l differences of the three molecules ($l_{PIC} < l_{PEN} < l_{DIP}$). While the mixing behavior is similar, the ordering behavior of the two systems differs. For both systems the size of coherently scattering islands derived from the (11l)-reflection decreases as the mixing ratio approaches 1:1. However, while it is reduced by a factor of 3 for PEN:PIC, the decrease in PEN:DIP is more than a factor of 13. For perfectly equimolar blends of PEN and DIP even a complete break-down of the long-range order in plane is expected Supplementary Reference [1]. We emphasize that this decrease in long-range order is only observed parallel to the substrate surface. Perpendicular to the substrate surface the long-range order is preserved in PEN:DIP blends and even improved compared to the pure films Supplementary Reference [1].

Supplementary Note 2

The changes in the PL spectra of PEN mixed with DIP and PIC with varying f_{PEN} are similar, but there are some distinct differences worth mentioning. The PIC spectra in Fig. 2 d) show a more abrupt shift to monomeric PEN emission than the DIP spectra. We believe these differences arise from the higher degree of orientational order in the PIC films. Since the herringbone angle is well-defined for all mixing ratios, the variation in the local molecular environment is less continuous than in the case of PEN:DIP blends. This becomes particularly important in the PL spectra, in which small contributions of PEN with another PEN molecule being its nearest neighbour can lead to a substantial decrease in the PL-intensity due to efficient SF and therefore, to a more abrupt change in the spectral shape. The replacement of nearest neighbours contributes also in blends of PEN:DIP. However, here there is an additional effect, namely, the decrease in long-range order parallel to the substrate surface. This leads to a possible variation of herringbone angles between translationally inequivalent molecules and a more disordered, but therefore, also more continuously changing local environment. This interpretation is supported by the fact that the PL spectrum changes continuously with the mixing ratio, indicating that the efficiency of fast decay channels such as singlet fission is reduced continuously rather than abruptly as it is the case in PEN:PIC blends.

Supplementary Note 3

The simulated absorption spectra and Davydov splitting (DS) calculations presented in Fig. 3 in the main text were evaluated from the eigenstates of a Holstein-like Hamiltonian that

includes Frenkel and charge-separated electronic excitations that are vibronically coupled to the dominant intramolecular vibrational mode. Complete details of the model can be found in Supplementary Reference [3]. Briefly, the crystals were modeled as two-dimensional sheets lying in the ***a-b*** herringbone plane and consisting of 20 unit cells along each of the crystalline ***a*** and ***b*** directions (800 total molecules). All intermolecular Coulombic (transition-dipole) interactions between Frenkel excitons were included. Electron and hole transfer between nearest-neighbor inequivalent and equivalent molecules was also considered. Finally, linear vibronic coupling to the 1380 cm^{-1} intramolecular vibrational mode was included by treating the nuclei in the excited states as shifted harmonic oscillators. The excited and ground state nuclear potential wells were assumed to be of identical curvature. A complete list of model parameters can be found in Supplementary Reference [3].

For the DS calculations, the model assumes a pentacene crystal with lattice parameters that depend on f_{PEN} (Supplementary Figure 1). For simplicity, all model parameters are kept fixed to those presented in Supplementary Reference [3] with the exception of the charge transfer integrals which sensitively depend on the intermolecular spacing. For each f_{PEN}, the charge transfer integrals (see Supplementary Table 1) were calculated within the dimer *fragment approach* as implemented in the Amsterdam Density Functional (ADF) package [Supplementary Reference 4] based on the lattice parameters in Supplementary Figure 1.

In this approach, the orbitals of the dimer are expressed as linear combination of molecular orbitals of the fragments that are obtained by solving the Kohn-Sham equations. Essentially, the site energies, ε_1 and ε_2, and the transfer integrals t_{12}, are obtained by computing the following matrix elements:

$\varepsilon_i = \langle \varphi_i \rangle$ and $t_{ij} = \langle \varphi_j \rangle$

Here, ϕ_i and ϕ_j correspond to the HOMO/LUMO orbitals of the isolated molecules (*i.e.* fragments). The transfer integral t_{12} has been evaluated at density functional theory (DFT) level using the B3LYP (Becke, three-parameter, Lee-Yang-Parr) hybrid functional [Supplementary Reference 5] with a Double Zeta basis set. However, due to the non-orthogonality of the fragment orbital basis set, the transfer integral value is not uniquely defined and depends on the definition of the energy of the origin [Supplementary Reference 6]. The problem is solved by applying a Löwdin transformation to the initial electronic Hamiltonian resulting in the following expression of the transfer integral

$$\tilde{t}_{12} = \frac{t_{12} - (\varepsilon_1 + \varepsilon_2) S_{12}}{1 - S_{12}},$$

where the parameter S_{12} represents the orbitals overlap.

<u>Supplementary note 4</u>

Using a pump wavelength of 400nm, where both, PEN and DIP absorb, led to a more complicated situation in which there are two pathways to triplet generation (see Supplementary Figure 6). First, direct excitation of the PEN leads to rapid SF, as with 600 nm excitation. But as the DIP concentration grows, a second pathway begins to play a role: excitation of the DIP followed by energy transfer to the PEN and subsequent SF. This second pathway will slow down as the DIP concentration increases, and thus we see a clear evolution with DIP loading, in which the total rate of fission slows down as it waits for energy transfer from the DIP molecules to the PEN molecules. The initial decay rates were independent of pump fluence, as expected because SF occurs more rapidly than exciton-exciton annihilation in crystalline PEN. This result is particularly interesting in view of the publication of Tian et al. [47], who studied blends of rubrene with spacer molecules and observed an increase in the SF time constant and who partially assigned this to energy transfer from the spacer molecules to rubrene.

Supplementary References

[1] A. Aufderheide, K. Broch, J. Novak, A. Hinderhofer, R. Nervo, A. Gerlach, R. Banerjee and F. Schreiber, Mixing-induced anisotropic correlations in molecular crystalline systems, Phys. Rev. Lett., 109, 156102, (2012).

[2] J. Dieterle, K. Broch, A. Hinderhofer, H. Frank, J. Novak, A. Gerlach, T. Breuer, R. Banerjee, G. Witte and F. Schreiber, Structural properties of picene-perfluoropentacene and picene-pentacene blends: Superlattice formation versus limited intermixing, J. Phys. Chem. C, 119, 26339, (2015).

[3] N. Hestand, H. Yamagata, B. Xu, D. Sun, Y. Zhong, A. Harutyunyan, G. Chen, H.-L. Dai, Y. Rao and F. C. Spano, Polarized absorption in crystalline pentacene: Theory vs. experiment, J. Phys. Chem. C, 119, 22137 , (2015).

[4] G. te Velde, F. M. Bickelhaupt, E. J. Baerends, C. Fonseca Guerra, S. J. A. van Gisbergen, J. Snijders and T. Ziegler, Chemistry with ADF, J. Comput. Chem., 9, 931, (2001).

[5] A. Becke, Density-functional thermochemistry. III. The role of exact exchange, J. Chem. Phys., 98, 5648, (1993).

[6] J. Huang and M. Kertescz, Validation of intermolecular transfer integral and bandwidth calculations for organic molecular materials, J. Chem. Phys., 122, 1234707, (2005).
